# An evaluation of bicycle-specific agility and reaction times in mountain bikers and road cyclists

**DOI:** 10.17159/2078-516X/2020/v32i1a8576

**Published:** 2020-01-01

**Authors:** K Buchholtz, T L Burgess

**Affiliations:** 1 Department of Physiotherapy, LUNEX University, Luxembourg; 2 Division of Exercise Science and Sports Medicine, Faculty of Health Sciences, University of Cape Town; 3 Division of Physiotherapy, Faculty of Health Sciences, University of Cape Town; 4 Centre for Medical Ethics and Law, Faculty of Medicine and Health Sciences, Stellenbosch University, Cape Town, South Africa

**Keywords:** exercise intensity, performance, bicycling

## Abstract

**Background:**

Cycling is a popular recreational and competitive sport with many health benefits but also significant risks, with 85% of recreational cyclists reporting an injury each season. The most common mechanism of injury is through a loss of control of the bicycle, and collisions with other objects. Reaction time and agility in cyclists may contribute to the ability to control a bicycle.

**Objectives:**

To evaluate bicycle-specific agility and reaction time in cyclists.

**Methods:**

The study was a cross-sectional observational study. Thirty-five cyclists (27 males, eight females) participated in this study. Participants attended a single testing session where they completed a bicycle-specific agility test, and online simple and choice reaction time testing while cycling at three different exercise intensities.

**Results:**

There was a significant difference in agility between males and females (p=0.01). There was also a significant difference in choice reaction time between cycling at ‘light’ and ‘very hard’ intensities (p=0.004), and a significant positive relationship between agility and simple reaction time at a ‘hard’ intensity.

**Discussion:**

Choice reaction time improved at ‘very hard’ cycling intensity, supporting the theory that increased exercise intensity improves cognitive arousal. This reaction time may be essential as a means to avoid collisions and falls from bicycles. Bicycle-specific agility appears to be related to simple reaction time, but there are no existing validated bicycle-specific agility tests available. The value of the tests undertaken by the authors needs to be assessed further.

**Conclusion:**

Choice reaction time was significantly decreased in high intensity cycling compared to cycling at low intensities. Further prospective studies are needed to establish links between reaction times and bicycle-specific agility.

Cycling is a popular recreational and competitive sport for people of all ages and has seen increases in participation worldwide. Johnson et al. identified an 11% increase in the cycling population of Australia from 2001 to 2007, and similar general trends have been observed in the United States of America and Europe ^[1]^. In South Africa, there are over 16 000 members registered with the Pedal Power Association, an association for recreational cyclists.

There are many health benefits to cycling whether as a sport for leisure, or as a means of transport. These benefits include improved cardiovascular endurance, greater muscle fitness, improved bone health, prevention of weight gain, and a lower risk of heart disease, stroke and high blood pressure ^[2]^. Despite all these benefits, cycling poses an injury risk due to its physical nature and exposure to external factors such as vehicles and obstacles ^[3]^.

Aleman and Meyers estimate that 85% of cyclists are injured during the cycling season in both mountain and road cycling ^[4]^. The most common mechanisms of acute injury in mountain biking are falls related to a loss of control of the bicycle, while road cyclists report collisions with other vehicles and bicycles ^[1,4]^. Cycling takes place in dynamic environments, thus there are several factors which are related to the risk of injury. Excessive fatigue, low level of cycling experience, inappropriate or improperly adjusted equipment, terrain, and conditioning and fitness levels are all factors that may increase a cyclist's injury risk ^[4]^.

One of these factors is reaction time (RT) ^[1]^. Johnson et al. found a significant association between cyclist RT, a post-event (post-collision or near collision with car) manoeuver, and the severity of this incident between cyclists and car drivers ^[1]^.

There are multiple types of RT, including simple, choice and discrimination. Simple RT is a single response to a single stimulus, and choice RT is a correct response to multiple random stimuli ^[5]^. Discriminatory or recognition RT is a correct response to multiple stimuli after determining whether a response is appropriate ^[5]^. Reaction time is affected by age, sex, physical activity and fatigue ^[6]^.

Previous studies have investigated the associations between reaction time and other variables during sport. Moradi and Esmaeilzadeh assessed speed, agility and reaction time and found that agility in schoolboys correlates with quicker RT ^[7]^. Intensity of the physical activity or the level of cognitive ‘arousal’ has also been linked to a faster RT ^[5]^. Skilled sportspersons have better cognitive function and quicker RT during submaximal physical activity ^[8]^. However, there is limited literature on reaction time and agility in cycling specifically, which warrants further investigation. The aim of this study was to evaluate bicycle-specific agility and reaction times in mountain bikers and road cyclists at different intensities of cycling.

## Methods

### Study design

This is a descriptive cross-sectional study.

### Participants

Thirty-five healthy male and female mountain bikers and road cyclists aged 18 to 69 years, who cycled a minimum of four hours per week over the past six months were recruited for this study. Participants were excluded if they reported any musculoskeletal injury in the six weeks prior to the study, or if they were considered to be high risk for physical activity on the Physical Activity Readiness Questionnaire (PAR-Q) ^[9]^.

### Ethical considerations

This study was approved by the Human Research Ethics Committee of the Faculty of Health Sciences, University of Cape Town (HREC REF: 210/2017). The study adhered to the ethical principles outlined by the Declaration of Helsinki (Fortaleza, Brazil, 2013).

### Procedure

Participants attended a single testing session at the Sports Science Institute of South Africa, Cape Town. Prior to inclusion in the study, they completed an informed consent form and the PAR-Q to assess for risk of adverse effects from physical activity ^[9]^. Once accepted into the study, the participants completed a self-developed questionnaire to assess their demographic and training history. The questionnaire was assessed before use in this study by a panel of experts for construct and content validity. Visual acuity was screened using a Snellen visual acuity chart to ensure sufficient vision to complete the testing. Body mass (kg), stature (m) and waist circumference (cm) were recorded, and body mass index (BMI) was calculated as kg/m^2^.

**Fig. 1:**
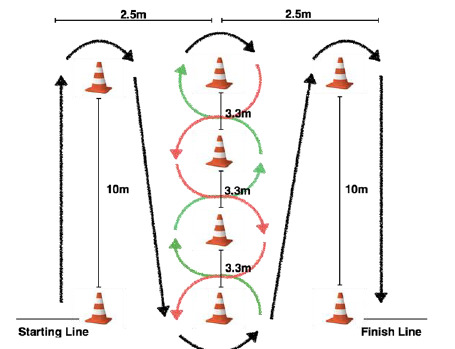
Modified Illinois Agility test (adapted from Raya et al, 2013) ^[10]^

A modified Illinois Agility Test (IAT) was used to assess bicycle-specific agility (Fig. 1) ^[10]^. Participants were instructed to cycle around cones on the floor as laid out in Fig. 1, as quickly as possible. The time was recorded in seconds. The test was terminated if participants stopped at any point during the test to regain balance, or if they placed a foot on the ground. Participants were allowed two practice runs of the test to ensure familiarity with the test. Participants completed the test three times, and the fastest time was recorded for analysis.

**Fig. 2:**
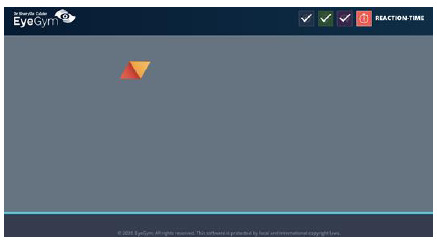
Screenshot of the reaction time programme

Reaction time was assessed at different intensities of exercise using a protocol previously described by Delignières et al. ^[8]^. The reaction time tests were conducted using an online programme (EyeGym) created by Dr Sherylle Calder to measure simple RT (SRT) and choice RT (CRT) through visual stimuli ^[11]^. In these tests, participants responded to an image appearing on a screen (Fig. 2). In the simple reaction time task, the participant responded as quickly as possible to a single item appearing on the screen. For choice reaction time, the participant would have multiple images on the screen but responded only to a single image, ignoring all the others. The reliability and validity of this online programme has not been previously established but is similar to the study by Delignières et al. who used joysticks to react to a stimulus on a computer screen ^[8]^. Reaction time was assessed with participants cycling on a Wattbike (Wattbike Ltd, Nottingham, United Kingdom) stationary trainer. The Wattbike was set up based on each individual participant’s height and comfort. A laptop was placed at eye level in front of the Wattbike and a keyboard placed on the handlebars.

Participants were instructed to cycle at three different intensities using the modified Borg Scale to determine the intensities. Participants warmed up for five minutes at an RPE of six (‘very, very light’ intensity). Simple RT and CRT were assessed at RPE levels of 11 (‘fairly light’), 15 (‘hard’) and 18 (‘very hard’). Participants cycled at each level for 10 minutes. Simple RT and CRT were assessed for five minutes into each level of intensity. Participants completed three SRT and CRT tests at each intensity, and the fastest SRT and CRT were recorded. Cadence and wattage were monitored during the test as an indication of effort between stages, but not used in analysis as the Wattbikes were not regularly calibrated.

### Statistical analysis

Descriptive statistical analyses were performed on the anthropometric data. Shapiro-Wilkes tests were performed for normality. Agility and reaction time results were found to be not normally distributed, and non-parametric tests were performed on these variables. T-tests were performed to assess differences between descriptive characteristics in male and female groups. Mann-Whitney U tests were performed to assess the difference between agility and reaction between male and female cyclists. Kruskal-Wallis tests were performed to assess differences between the road cycling, mountain biking and both groups. No post-hoc tests were performed as there were no significant results. A Friedman’s ANOVA was performed with Wilcoxon signed rank test to determine the difference between RT at different intensities of cycling. Associations between variables were assessed using a Spearman’s correlational analysis. Statistical significance was accepted as p < 0.05.

## Results

Thirty-five participants completed the testing protocol. Their descriptive characteristics are reported in Table 1. Body mass, stature and waist circumference were significantly greater in male participants (p<0.05).

**Fig. 3:**
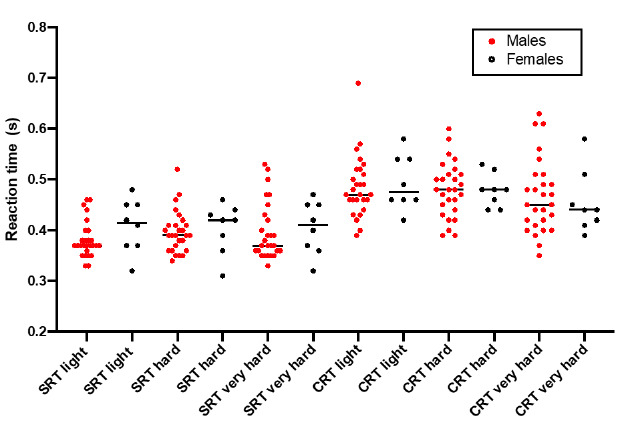
Individual results of simple and choice reaction time at light, hard and very hard intensities in males and females

The mean years cycled was 21±13 years. The average distance cycled per week was 150±109 km. Eight participants were road cyclists (23%), two participants were mountain bikers (6%) and 21 participants cycled in both disciplines (60%). Four participants did not complete the questionnaire section on the cycling discipline. Individual results for simple and choice reaction time at light, hard and very hard intensities for males and females are presented in Fig. 3.

There were no significant differences in SRT or CRT between sexes (Table 2). There was a significant difference in agility between males and females (U=46.0, *p=0.01*) (Table 2). There were no significant differences in SRT at different intensities of cycling for all participants (Table 3); however, there was a significant difference between CRT at different intensities (ANOVA chi-sq=10.93, *p=0.004*). With the use of post-hoc testing, this difference was identified between CRT at ‘light’ intensity and ‘very hard’ intensity (Z=3.23, *p=0.004*), but not between ‘light’ and ‘hard’, or ‘hard’ and ‘very hard’ intensities.

There was significant, but weak positive correlation between bicycle-specific agility and SRT at a ‘hard intensity’ (r=0.38, *p=0.026*). There were no other significant relationships between RT and agility (Table 4).

## Discussion

Sex-related differences in bicycle-specific agility scores were consistent with findings observed using standard Illinois agility tests in other studies ^[12]^. Agility is a complex task incorporating acceleration, deceleration, change of direction and decision-making ^[13]^. Reasons for sex-related differences may include greater muscle mass, greater aerobic capacity and higher anaerobic thresholds as a consequence of genetic and hormonal differences ^[14]^.

In this study, the authors did not observe any sex-related differences in SRT and CRT contrary to the findings in multiple studies as reported by Kosinsky ^[5]^. This is most likely due to the small number of females in the study. The authors identified a significant reduction in CRT at ‘very high’ levels of cycling intensity compared to ‘light’ intensity. Reaction time has been found to improve with intermediate levels of arousal, and to be lower when either too relaxed or too tense in other studies ^[5]^. Submaximal levels of physical activity create the optimal arousal levels required for maximal cognitive functioning and potentially, RT ^[15]^. Previous research has demonstrated improvements in choice RT for up to eight minutes after exercise ^[5]^.

This study identified significant positive relationships between bicycle-specific agility and STR at a ‘hard’ intensity. This is supported by Moradi and Esmaeilzadeh who found significant associations between running agility and SRT ^[7]^. Bicycle-specific agility differs in that it has an additional piece of equipment with different capabilities in terms of change of direction and which has not as yet been investigated as a valid clinical assessment.

### Limitations

During testing, it was noted that the keyboards used for RT testing lacked the sensitivity needed in fast physical testing. The same keyboards were used throughout the study, which may have provided limited potential equipment bias. Thus, the results may not be as accurate as required to adequately assess reaction time. The reaction time tests have not been previously validated, and this should be a priority in further research using these tests. The small participant groups when analysing results between males and females and between the types of cycling may have limited the statistical power in these results.

## Conclusion

Choice RT was significantly improved in response to high intensity cycling compared to the low intensities. Quicker SRT was related to faster bicycle-specific agility performance. Further prospective studies are needed to establish links between reaction times, bicycle-specific agility and control of the bicycle in cyclists.

## Figures and Tables

**Table 1:** Descriptive characteristics of male and female participants

Variable	Male (n=27)	Female (n=8)	t-value	p-value
**Age (years)**	44.3±16.8	43.1±14.2	0.16	0.875
**Body mass (kg)**	81.5±14.8	61.4±14.3	3.32	0.0002*
**Stature (cm)**	177.5±7.0	163.4±7.3	4.95	0.0002*
**Body mass index (kg/m** **^2^** **)**	25.7±3.9	22.9±4.3	1.75	0.089
**Waist circumference (cm)**	87.4±10.1	74.4±8.7	3.20	0.004*

Data are expressed as mean ± SD.

* indicates p<0.05.

**Table 2:** Results of a Mann-Whitney U test comparing agility and reaction time scores between male and female participants

Variable	Group	N	Median (IQR)	Sum of	U-value	p-value
**Agility**	Male	27	29.2 (27.2–32.1)	425	46.0	0.01*
	Female	8	33.7 (32.1–37.0)	206		

**Simple Reaction: light intensity**	Male	27	0.37 (0.36–0.40)	451	72.5	0.16
	Female	8	0.42 (0.37–0.45)	180		

**Simple Reaction: hard intensity**	Male	27	0.39 (0.36–0.41)	463	84.5	0.37
	Female	8	0.42 (0.37–0.44)	168		

**Simple Reaction: very hard intensity**	Male	27	0.37 (0.36–0.43)	470	92.0	0.54
	Female	8	0.41 (0.36–0.45)	160		

**Choice Reaction: light intensity**	Male	27	0.47 (0.46–0.52)	477	98.5	0.72
	Female	8	0.48 (0.46–0.54)	154		

**Choice Reaction: hard intensity**	Male	27	0.48 (0.44–0.51)	488	106.5	0.96
	Female	8	0.48 (0.45–0.51)	143		

**Choice Reaction: very hard intensity**	Male	27	0.45 (0.41–0.51)	497	97.0	0.68
	Female	8	0.44 (0.41–0.50)	133		

Data are presented as median (interquartile range).

* indicates p<0.05.

**Table 3:** Relationship between simple and choice reaction time at light, hard and very hard intensities, with post hoc testing

	Friedman’s ANOVA		Dunn’s post hoc					

	Friedman statistic	p-value	Light vs. hard (z-value)	p-value	Light vs. very hard (z-value)	p-value	Hard vs. very hard (z-value)	p-value
**SRT**	1.44	0.488	-	-	-	-	-	-
**CRT**	10.93	0.004	1.434	0.455	3.227	0.0038*	1.793	0.219

* indicates p<0.05.

SRT, simple reaction time; CRT, choice reaction time.

**Table 4:** Correlations between agility and simple and choice reaction time at light, hard and very hard intensities

Variable	r-value	p-value
**Simple Reaction: light intensity**	−0.06	0.748
**Simple Reaction: hard intensity**	0.38	0.026*
**Simple Reaction: very hard intensity**	0.31	0.067
**Choice Reaction: light intensity**	0.13	0.456
**Choice Reaction: hard intensity**	0.33	0.055
**Choice Reaction: very hard intensity**	0.27	0.123

* indicates p<0.05.
